# Increasing Cathode Potential of Homogeneous Low Voltage Electron Beam Irradiation (HLEBI) to Increase Impact Strength of Carbon Fiber Reinforced Polycarbonate and Characterization by XPS C1s and O1s Peaks

**DOI:** 10.3390/ma18235471

**Published:** 2025-12-04

**Authors:** Fumiya Sato, Kouhei Sagawa, Helmut Takahiro Uchida, Hirotaka Irie, Michael C. Faudree, Michelle Salvia, Akira Tonegawa, Satoru Kaneko, Hideki Kimura, Yoshitake Nishi

**Affiliations:** 1 Graduate School of Engineering, Tokai University, 4-1, Kitakaname, Hiratsuka 259-1292, Japan; stfmy0901@gmail.com (F.S.); helmutuchida@tokai.ac.jp (H.T.U.); ih2120@star.tokai-u.jp (H.I.); kimura@tokai.ac.jp (H.K.); west@tokai.ac.jp (Y.N.); 2 Doctoral Graduate School of Science & Technology, Tokai University, 4-1, Kitakaname, Hiratsuka 259-1292, Japan; atone@tokai.ac.jp; 3 Faculty of Liberal Arts and Science, Tokyo City University (TCU), 3-3-18, Ushikubo West Tsuzuki-ku, Yokohama 224-8551, Japan; 4 Laboratory of Tribology and Dynamics of Systems (LTDS), Ecole Centrale de Lyon (ECL), Cedex, 69134 Ecully, France; michelle.salvia@ec-lyon.fr; 5 Kanagawa Institute of Industrial Science & Technology (KISTEC), 705-1, Shimoimaizumi, Ebina 243-0435, Japan; kaneko@kistec.ac.jp

**Keywords:** thermoplastics, polycarbonate, carbon fiber, impact strength, cathode potential, electron beam irradiation, penetration depth, X-ray Photoelectron Microscopy (XPS)

## Abstract

In an interlayered carbon fiber reinforced polycarbonate (CFRPC) composite constructed of nine CF plies alternating between ten PC sheets, designated [PC]_10_[CF]_9_, applying homogeneous low voltage electron beam irradiation (HLEBI) at 200 kV cathode potential, with *V*_c_ setting at a 43.2 kGy dose, to both finished sample surfaces resulted in a 47% increase in Charpy impact strength and *a*_uc_ at median fracture probability (*P*_f_) of 0.50 over that of untreated, from 118 kJm^−2^ to 173 kJm^−2^. Increasingly higher *V*_c_ settings of 150, 175, and 200 kV successively increased *a*_uc_ at median-*P*_f_ of 0.50 to 128, 155, and 173 kJm^−2^, respectively. Strengthening is attributed to increasing the HLEBI penetration depth, *D*_th_, into the sample thickness. Since the [PC]_10_[CF]_9_ has an inhomogeneous structure, *D*_th_ is calculated for each ply successively into the thickness. Scanning electron microscopy (SEM) photos showed a hierarchy of fracture mechanisms from poor PC/CF adhesion in untreated; to sporadic PC adhesion with aggregated CF at 150 kV; to high consolidation of CFs by PC at 200 kV. X-ray photoelectron spectroscopy (XPS) examination of the CF surface in the fracture area showed C1s carbonate O–(C=O)–O and ester O–(C=O)–R peak generation at 289 to 292 eV to be non-existent in untreated; well-defined at 150 kV; and increased in intensity at 200 kV, after which a reduction was observed at 225 kV. Moreover, the 200 kV yielded the largest area sp^3^ peak at 49.5%, signifying an increase in graphitic edge planes in the CF, apparently as dangling bonds, for increased adhesion sites to PC. For O1s scan, 200 kV yielded the largest area O–(C=O)–O peak at 34%, indicating maximum PC adhesion to CF. At the higher 225 kV, increase in *a*_uc_ at *P*_f_ of 0.50 was less, to 149 kJm^−2^, and XPS indicated a lower amount of O–(C=O)–O groups, apparently by excess bond severing by the higher *V*_c_ setting.

## 1. Introduction

Carbon fiber reinforced polymer (CFRP) composites, in general, have been used for numerous applications due to their superior strength and stiffness while being lightweight to save on fuel consumption. Parts include sports equipment such as bicycle frames and snowboards, automobile parts such as chassis and brake parts, and dream-worthy aerospace vehicles. Aerospace applications include giant wings for the Boeing 777X and A380 Airbus and launch and re-entry vehicles and satellites [1]. Mars exploration vehicles have CFRP parts, such as the Curiosity Rover’s protective shield and the long CFRP propellers of Perseverance helicopter [2].

Carbon fiber reinforced thermoset (CFRTS) composites, particularly epoxies, have seen wide application, although a serious drawback is their non-recyclability. Various types of strengthening methods for CFRTS are covered here. Pre-stressing of long continuous fiber CF, used to generate compressive strength in epoxy polymer matrix, has been reported to increase both impact resistance and flexural strength in beam structures [3,4,5] and increase tensile strength in unidirectional CFRTS [6,7]. Adjusting the CF configuration, for example, ultra-thinning CF plies, in a unidirectional CFRTS laminate was found to lower fiber deflection and resin rich areas, improving mechanical properties [8], while unidirectional CF orientation at zero degrees was found to increase longitudinal stress from high velocity impact over that at 90 degrees [9]. To strengthen interlaminar strength in the *z*-direction of CFRTS laminates, co-woven carbon–liquid crystal polymer (CLCP) intralaminar hybrid composites [10] and Z-pinned composites have been engineered [11,12,13]. Various treatments for strengthening the interphase at the TS/CF interface have been reported [14] including plasma treatment [15,16,17,18,19,20], high energy irradiation [21,22,23,24], nickel surface coating [25,26,27], thermal treatment [28], building surface roughness [29], electro-polymer coating [30,31], sizing [32], electrochemical modification [33,34], and electro-spinning [35]. Also included have been acidic modification [36,37,38], aqueous ammonia treatment [39], ion addition [21], rare earth metal modification [40,41], graphene oxide (GO) dip coating [42], carbon nanotubes (CNTs) [43,44,45,46], controlling CNT orientation and length [47], and different procedures to deposit multi-walled CNTs (MWCNTs) on the CF surface [48,49,50]. In addition, modifying epoxy resin itself, for instance, novolak epoxy resin mixed with anime hardener and deposited on CFs for a 3D printing filament, produced a CFRTS composite with high glass transition temperature and tensile and flexural strength [51].

However, although CFRTSs are widely used for heavier load-bearing parts, the non-recyclability and inability to melt due to their crosslinked molecular network makes them extremely harmful to the environment. CFRTSs are usually incinerated or discarded in landfills.

A viable alternative has been the use of thermoplastic (TP) matrices since they are recyclable and can be repeatedly melted and solidified. However, a major drawback with carbon fiber reinforced thermoplastic (CFRTP) composites has been their significantly lower mechanical strength than their epoxy CFRTS counterparts. This is due to both PC and CF being non-polar and having hydrophobicity, poor wettability, and surfaces that are chemically inert, rendering adhesion difficult.

To strengthen the recyclable CFRTPs while accelerating Sustainable Development Goals (SDGs), a wide body of research has been carried out, mostly on treatments to CF to strengthen the TP/CF interface. These have included the following: investigating sizing agents [52,53,54]; increasing CF wettability by polymer colloid dispersion [55]; polymer compatibilizer coatings [56]; applying MWCNTs [57]; electrochemical treatment [33]; acid treatment [58]; plasma activation [59]; cold remote nitrogen oxygen plasma [60]; hybrid methods such as cryo-treatment, plasma, and acid [61]; γ-ray irradiation [24]; and low energy electron beam [62]. Modifying the TP matrix by blending to increase mechanical properties has also been carried out [63]. This list is not all-inclusive.

One of the widely used TPs has been the PC polymer with its recyclability, easy moldability and reforming, high impact strength, good electrical insulation, heat and flame resistance, and transparency. PC is typically used for electronic components, 3D printing, building construction materials, and aircraft windows, to name a few. Although the price of PC in September 2025 (USD 1.94/kg, Northeast Asia) [64] is reported to be close to that of epoxies (USD 1.98/kg, Northeast Asia)) [65], the recyclable PC has a solidification time which is much lower, about 1/10 that of epoxies, to reduce waste and energy consumption.

To increase the mechanical strength and other desirable properties of CFRPs, applying HLEBI has been one of the useful tools that works by activating the formation of terminated atoms, known as “dangling bonds, (DBs)” [66,67]. DBs are severed bonds of lone pair electrons that can strengthen materials by (1) creating internal compressive stress sites by repulsive forces between lone pair electrons as in CF or PC bulk, and (2) creating stronger adhesion between materials by bonding of DBs as lone pair electrons at the surface as at the PC/CF interface in carbon fiber reinforced thermoplastic (CFRPC). Although an excess HLEBI dose can generally decay materials by radiation damage, the optimum dose has been found to generate a strong adhesion force with covalent bonding at the CF/PC interface [68]. However, increasing the strength of CFRTPs to that of epoxy CFRTS remains a serious challenge. For the property of ultimate tensile strength (UTS) (*σ*_b_), untreated CFRPC (95.0 MPa) is reported to be 23% that of untreated epoxy CFRTS at 413 MPa, as shown in Table 1 [69]. The lower strength is attributed to the easy CF pullout due to the low wettability and insufficient bonding sites of PC with CF by their inertness, while epoxies have increased bonding site density, with higher wettability on CF [69]. To increase bonding site density, applying HLEBI directly to the CFs prior to dipping in PC has raised UTS to 290 MPa, closer to 70% of that of epoxy [68,69], as shown in Table 1.

Up to now, HLEBI has been successful in improving the material and physical properties of numerous materials, such as wetting and mist resistance [71,72], adhesion of glass fibers to polymers [73,74], adhering metal/polymer joints [75,76] and metal/CFRP joints [77], and increasing the strength of polymer/CF interlayered composites [66].

HLEBI at the low *V*_c_ of 170 kV is reported to strengthen the CF [78,79] and PC resin itself [80]. Interestingly, applying 1.12 MGy HLEBI at 170 kV has increased the tensile strength of CF to above 10 GPa [78], while PC impact strength at a median fracture probability (*P*_f_) of 0.50 has been reported to max out at 29.8 kJm^−2^ by applying 129 kGy (170 kV) HLEBI, above which significant reduction occurs [80]. DBs by the HLEBI has been proven to be a cause of strength enhancement in both CF [78,81] and PC [80] as evidenced by peaks generated by electron spin resonance (ESR) imaging of HLEBI-treated samples.

As for strengthening CFRPC by HLEBI, as far as the authors know, outside of our research group there has been no or little literature found [66]; therefore, our research up to now [66,80,81,82] is summarized in Table 2, with this study also listed. For the interlayered [PC]_4_[CF]_3_ composite composed of three CF plies alternating between four PC plies, applying HLEBI at *V*_c_ of 170 kV at 216 kGy dose to CF plies prior to lamination assembly and hot press is reported to increase the statistically lowest Charpy impact strength, *a*_uc_ (*a*_s_ at *P*_f_ = 0) 6% [66]. However, higher doses of 302 or 432 kGy to CF degraded the weakest [PC]_4_[CF]_3_ samples in their data sets, possibly due to PC ply damage by excess dose [80]. For the [PC]_4_[CF]_3_, the same level, 216 kGy, of HLEBI treatment was found to increase bending strength; *σ*_b_ at median-*P*_f_ = 0.50 25% [81]. Charge transfer from the CF to PC apparently occurs, creating DBs that bond and strengthen the PC/CF interface, along with strengthening the CF and PC components [66,81,82]. Another study showed that when an interlayered [PC]_10_[CF]_9_ composite was HLEBI-treated at *V*_c_ of 250 kV at doses from 86 to 172 kGy to finished specimen surfaces, the mean *a*_uc_ at *P*_f_ = 0.50 was increased up to 25 to 30% over those of untreated [82]. The HLEBI to the finished specimen surfaces, instead of every CF ply before assembly, lowers the number of steps and required energy use.

Therefore, the goal of this study is to obtain a further increase in *a*_uc_ of the [PC]_10_[CF]_9_ by setting *V*_c_ to intermediary values of 150, 200, and 225 kV, at the low dose of 43.2 kGy to prevent excess damage to the PC component. The novel part of this study is using HLEBI to increase the strength of the CFRPC since, presently, few studies are found in the literature. For maximum safety, utmost care to attain optimum HLEBI settings is highly recommended for each situation since excess *V*_c_, dose, or a combination thereof can weaken the composite.

## 2. Experimental Procedure

### 2.1. Composite Fabrication

**Figure 1:**
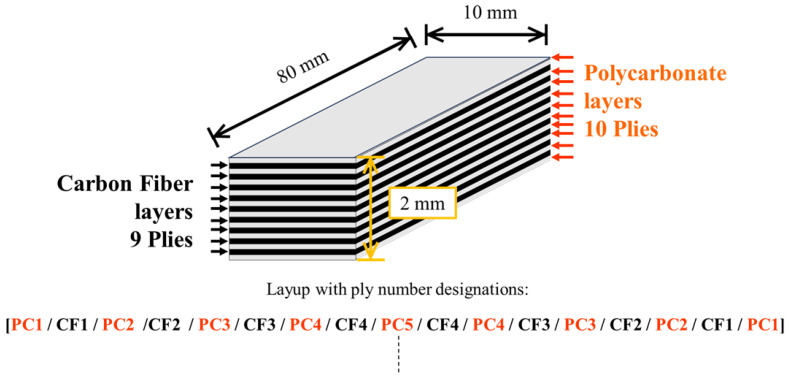
Schematic drawing of [PC]_10_]CF]_9_ CFRPC laminate sample showing dimensions (not to scale) and layup with ply number designations. Dashed line represents centerline of thickness.

The CFRPC (the 55.3 vol% CF) samples were prepared in Formosa (TAIRYFIL [FTC:EC3C], Formosa Plastics Co., Formosa, Kaohsiung City, Taiwan), with sized plain cross-weave CF plies (TR3110M, Mitsubishi Rayon Ltd., Tokyo, Japan) 230 µm in thickness prior to molding. Areal weight of CF was 198 to 200 gm^−2^ [83]. TP PC sheets (110 mm × 170 mm × ~286 µm) (Sugawara Kougei Ltd., Tokyo, Japan) were fabricated by hot-pressing PC particles (3 g) under 15 MPa at 418 Kfor 3 min. As illustrated in Figure 1, specimens were assembled as 9 CF plies alternating between 10 PC sheets (19 plies total), designated “[PC]_10_[CF]_9_”, then hot-pressed at 2.45 MPa at 508 K for 15 min. Sample dimensions were cut into 10 × 2.0 × 80 mm dimensions prior to HLEBI. It is assumed that after hot press, PC and CF layer thicknesses were 79 μm and 139 μm, respectively. As shown, outer plies going into the thickness from both sides will be designated as follows: “PC1”, “CF1”, “PC2”, and “CF2”. Samples were unnotched.

### 2.2. Conditions of HLEBI

An electron-curtain processor: Type CB 250/30/20 mA, Energy Science Inc. Woburn, MA, USA, Iwasaki Electric Group Co., Ltd., Tokyo, Japan) [66]. Samples were homogeneously irradiated by the linear electron beam gun with low energy through a titanium thin film window (thickness, *T*_Ti_ = 10 μm) attached to a 240 mm diameter vacuum chamber. Distance between the sample position and window was *T*_N2_ = 25 mm. A tungsten filament in a vacuum was used to generate the electron beam at a low energy condition, with irradiating current density (*I*) of 0.089 A·m^−2^ for one operation time. Although the electron beam is generated in a vacuum, the irradiated sample was kept under protective N_2_ gas with a residual concentration of O_2_ gas below 300 ppm. The constant flow rate of N_2_ was set to be 1.5 L/s at 0.1 MPa N_2_ pressure. Each irradiation dose was applied for only a short time (0.23 s) to avoid excessive heating of the sample; the temperature of the sample surface remained below 323 K just after irradiation. The sample in the aluminum plate holder (15 cm × 15 cm) was transported on a conveyor at a constant speed of 10 m/min. The sheet electron beam irradiation was applied intermittently: one sweep going one way was 0.0432 MGy. Repeated irradiations were used to increase total irradiation dose. The interval condition of 30 s was applied between each sweep. Total irradiation dose, *D*, is proportional to irradiation current (*I*, mA) and number of irradiations (*N*), whereas it is inversely proportional to conveyor speed (*S*, m/min). Irradiation dose is controlled by integrated irradiation time in each sample. Here, irradiation dose was corrected by using a nylon dosimeter of RCD radiometer film (Far West Technology, Inc. 330-D South Kellogg, Goleta, CA, USA) Model FWT-60-00, with an irradiation reader, Model FWT-92D.

To investigate effects of *V*_c_ on Charpy impact strength, *a*_uc_, both sides of finished [PC]_10_[CF]_9_ samples were treated with HLEBI at cathode potential, with *V*_c_ settings at 150, 175, 200, or 225 kV, and compared with untreated. HLEBI dose, *D*, was held constant at 43.2 kGy. Five samples were tested at each experimental condition as listed in Table 3.

### 2.3. Penetration Depth, D_th_, and Calculations for Interlayered Samples

When *V*_c_ (kV) is defined as surface electrical potential, and *ρ* (kg·m^−3^) is density, *D*_th_ (m) of HLEBI is expressed by the Christenhusz and Reimer equation [84]:

*D*_th_ = 66.7 *V*_c_^5/3^/*ρ* (1)

**Figure 2:**
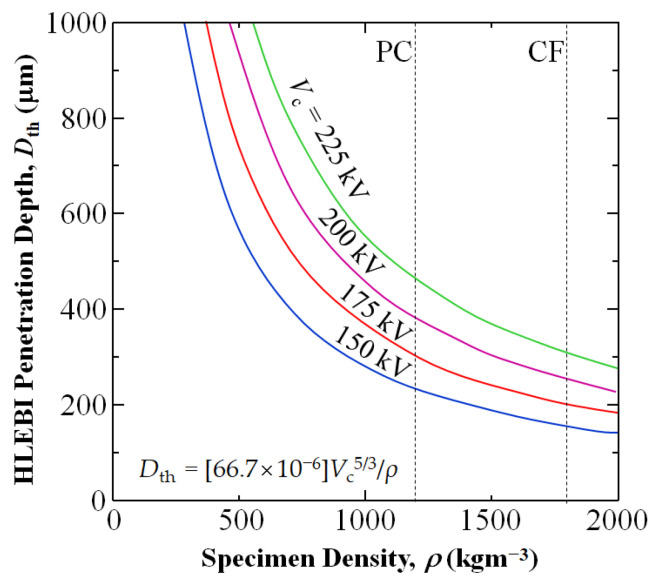
Change in calculated HLEBI penetration depth, *D*_th_ (μm), as a function of specimen density, *ρ* (kgm^−3^). Densities of PC and CF are indicated by horizontal lines.

Figure 2 shows calculated plots of *D*_th_ vs. *ρ* [84] for *V*_c_ of 150, 175, 200, and 225 kV with vertical lines indicating *ρ* of PC and CF, whose intersections yield *D*_th_ values.

The *D*_th_ values are calculated for each successive layer into the [PC]_10_[CF]_9_ sample thickness, assumed as an inhomogeneous interlayered structure, beginning with the outer PC1 plies. Using the principal form of Equation (1), *V*_c_ is decreased to surface electrical potential at the PC1 ply, *V*_x(PC1)_, where *x* = *V*_c_, by dropped potentials from passing through the Ti window, Δ*V*_Ti_, and N_2_ protective gas atmosphere, Δ*V*_N2_, respectively [80]:

*V*_x(PC1)_ = *V*_c_ − Δ*V*_Ti_ − Δ*V*_N2_ (2)

When *V*_c_ is set to 150 kV, *V*_x(PC1)_ becomes *V*_150(PC1)_. Given that the densities of Ti (*ρ*_Ti_) and N_2_ (*ρ*_N2_) are 4540 kgm^−3^ and 1.13 kgm^−3^, respectively, Δ*V*_Ti_ of 24.1 kV and Δ*V*_N2_ of 16.9 kV are obtained in Equations (3) and (4) [80]:

Δ*V*_Ti_ = *T*_Ti_/D_thTi_ × *V*_c_ = *T*_Ti_*ρ*_Ti_/[66.7 × (*V*_c_)^2/3^] =(10^−5^ m) × (4540 kgm^−3^)/[66.7 × (*V*_c_)^2/3^] = 24.1 kV (3)

Δ*V*_N2_ = *T*_N2_/D_thN2_ × *V*_Ti_ = *T*_N2_*ρ*_N2_/[66.7 × (*V*_Ti_)^2/3^] = (25 × 10^−3^ m) × (1.13 kgm^−3^)/[66.7 × (*V*_c_ − Δ*V*_Ti_)^2/3^] = 16.9 kV (4)

The surface potential at PC1 is

*V*_150(PC1)_ = 150 kV − 24.1 kV − 16.9 kV = 109.0 kV (5)

Therefore, *D*_th_ of HLEBI into PC1 is calculated from the density of PC, *ρ*_PC_ (1200 kgm^−3^):

*D*_th_ = 66.7 *V*_150(PC1)_^5/3^/*ρ* = 138 μm (6)

This is greater than the 75 μm thickness, indicating full penetration. Table 4 lists *D*_th_ values into PC1 for *V*_c_ settings of 150, 175, 200, and 225 kV, along with their Δ*V*_Ti_, Δ*V*_N2_, and *V*_x(PC1)_ values, respectively, from Equations (1)–(6). As expected, *D*_th_ values increase with *V*_c_. Since all *D*_th_ values are greater than the 75 μm PC1 thickness, HLEBI will penetrate into the next ply, CF1. Therefore, to estimate *D*_th_ into CF1 and PC2, the same procedures of using Equation (3) (or Equation (4)) and Equation (1) are employed (see Section 4).

### 2.4. Charpy Impact Test

The Charpy impact test was chosen since it is regarded as a primary test to evaluate structural materials used in airplanes, space vehicles, and automobiles to provide information on reaction and resistance to high velocity impact, such as bird-strike, hailstones, or volcanic rock, for maximum safety [85,86]. Conventionally, the impact test for CFRP laminates is conducted with a hemispherical impactor as a slow point force to the center of a square or rectangular specimen ~70 to 150 mm^2^ [52] by drop tower or projectile [87,88,89,90]. This is followed by non-destructive testing (NDT), such as by ultrasonic sensor, to detect damage undetectable to the eye, for example, ply delamination. Subsequently, mechanical testing, for instance, compression after impact (CAI), or fatigue, is typically carried out on the laminates for safety design [91,92].

On the other hand, the Charpy impact test utilizes a drop weight pendulum to determine impact absorption mechanisms and is used to evaluate the impact toughness of materials for quality control (QC). We do not claim Charpy impact to be a replacement for point impact followed by CAI. Rather, Charpy can be used as a quick and inexpensive way to investigate the reaction of CFRP to high velocity impact. This is to screen new candidate materials for aircrafts or automobiles for possible extensive testing, such as CAI, tensile, open hole compression, shear, fatigue, and environmental such as ultraviolet rays, temperature, and humidity. One reviewer suggested that micro-hardness tests should be conducted for the [PC]_10_[CF]_9_. Vickers hardness is traditionally employed for homogeneous materials such as metals and neat resins, but the 2-phase PC–CF system poses many challenges such as inhomogeneous CF distribution, small CF diameter, and large indenter, making it difficult to obtain clear and reliable indentations. In addition, tensile and shear tests are highly required for selecting new, strong materials, but are beyond the scope of this study. Hence, we focus on the Charpy impact test for the [PC]_10_[CF]_9_ specimens.

Charpy impact values (*a*_uc_) of the [PC]_4_[CF]_3_ samples were obtained using a standard impact fracture energy measurement system (Shimadzu Corporation, Tokyo, Japan, Model No.51735) according to Japanese industrial standard (JIS) K 7077 [93]. The impact fracture energy, *E* (kJ), is expressed by the following [94]:

*E* = *WR*[(cos*β* − cos*α*) − (cos*α*’ − cos*α*)(*α* + *β*)/(*α* – *α’*)] (7)

Here, *E*, *W*, *R*, *β*, *α*, and *α’* are impact fracture energy (kJ), hammer mass (0.86 kg), length (0.25 m) of hammer weight point from rolling center, maximum angle (150°) after impact (Radians), start angle before impact (*α* = 2.3 Radians or 150°), and maximum angle of the blank test, respectively. Usually, three blank tests are performed and the average is taken to calibrate the instrument for temperature, humidity, and atmospheric pressure. The Charpy impact value (kJm^−2^) is expressed by the following [74]:

*a*_uc_ = *E*/(*b* × *t*) (8)

Here, *E*, *b* (=10 ± 0.2 mm), and *t* (=2 ± 0.15 mm) are impact fracture energy (J), sample width (mm), and thickness (mm). The distance between supporting points was 80 mm.

Evaluating the *P*_f_ has been a convenient method of quantitatively analyzing experimental values relating to fracture, often used in industry to determine manufacturing reliability in QC. *P*_f_ is expressed by the following equation, which is a generalized form of the median rank method [80]:

*P*_f_ = (*I* − 0.3)/(*N*_s_ + 0.4) (9)

where *N*_s_ and *I* are the total number of samples and the ascending strength order of each sample, respectively. Impact tests were carried out 30 ± 0.5 h after HLEBI treatments.

### 2.5. Scanning Electron Microscopy (SEM) and Energy Dispersive X-Ray Spectroscopy (EDS)

A tabletop SEM Model JCM-6000 Plus Neoscope (JEOL, Ltd., Tokyo, Japan) was used to analyze fracture surfaces of the samples at magnification of 600×, with acceleration voltage of 15.0 kV, and sweep count of 50. Elemental analysis scans for oxygen (O) and carbon (C) were performed by the energy dispersive X-ray spectroscopy (EDS) component in the SEM JCM-6000 with 360 deg tilt rotation motor, and 2-axis motor-driven stage in *x* and *y* directions. Win 7 EDS elemental analysis software was used.

### 2.6. X-Ray Spectroscopy (XPS)

XPS tests were conducted on CF surface at the fracture areas of the samples using a Model PHI Quantera II XPS instrument from ULVAC-PHI, Inc., Chigasaki, Kanagawa, Japan.

## 3. Results

### 3.1. Impact Strengthening

**Figure 3:**
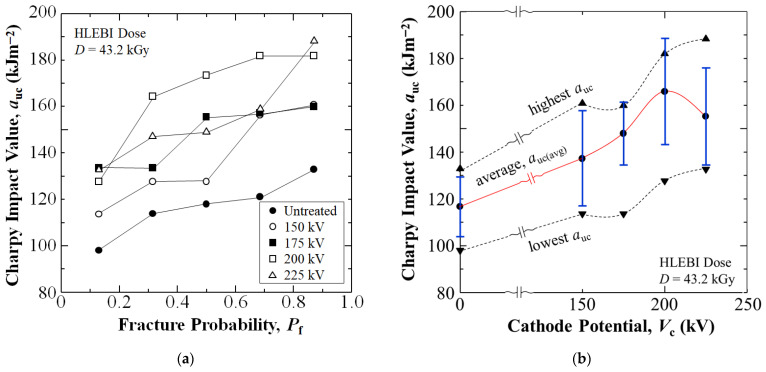
Changes in Charpy impact value, *a*_uc_ (kJ/m^2^) vs. (**a**) *P*_f_ for untreated and HLEBI data sets; and (**b**) *a*_uc_ vs. cathode potential, *V*_c_, respectively, of the [PC]_10_[CF]_9_ samples. In (**b**), the red curve indicates *a*_uc,avg_ values; dotted lines indicate highest and lowest *a*_uc_ values; and blue bars indicate standard deviations. HLEBI dose was 43.2 kGy for all HLEBI-treated samples.

Figure 3a shows that when applying a 43.2 kGy HLEBI dose to [PC]_10_[CF]_9_ finished sample surfaces, the *V*_c_ setting of 200 kV increases Charpy impact value, *a*_uc_ (at median-*P*_f_ of 0.50), 47% over that of untreated, from 118 kJm^−2^ to 173 kJm^−2^. In addition, Figure 3a shows that increasingly higher *V*_c_ settings of 150, 175, and 200 kV successively increase *a*_uc_ at median-*P*_f_ of 0.50 from 118 kJm^−2^ for untreated to 128, 155, and 173 kJm^−2^, respectively. However, the higher 225 kV *V*_c_ setting results in a decrease in *a*_uc_ at *P*_f_ of 0.50 to 149 kJm^−2^.

Figure 3b shows that the 200 kV setting raises the average *a*_uc_ (*a*_uc,avg_) (red curve) 42% from 117 to 166 kJm^−2^. Moreover, the 200 kV setting increases the highest *a*_uc_ value (*P*_f_ = 0.87) 37% from 133 to 182 kJm^−2^ and lowest *a*_uc_ value (*P*_f_ = 0.13) 30% from 98 to 128 kJm^−2^. Therefore, for the *V*_c_ settings tested, 200 kV appears to be optimum for *a*_uc_.

### 3.2. Fracture Surface Observation by SEM and EDS

**Figure 4:**
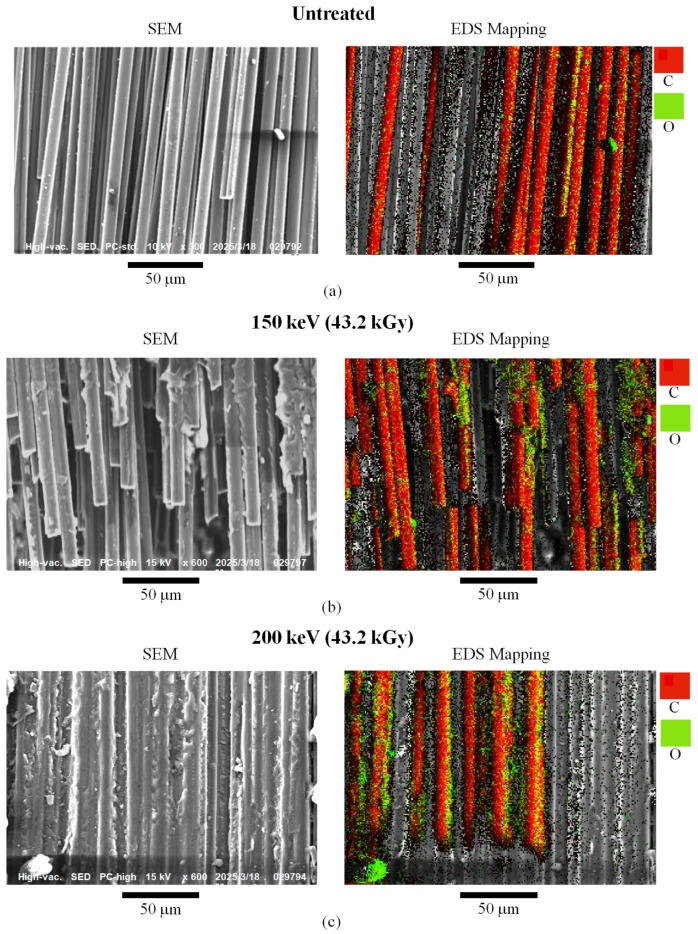
SEM photographs (**left**) and EDS mappings (**right**) of fracture surface of {PC}_10_[CF]_9_ samples: (**a**) untreated; and HLEBI-treated by 43.2 kGy dose at *V*_c_ setting of (**b**) 150 kV and (**c**) 200 kV.

Figure 4a–c, left side, shows the SEM of the fracture surfaces of untreated, 150, and 200 kV samples (43.2 kGy), respectively. A hierarchy of strengthening mechanisms is apparent in Figure 4 with increasing *V*_c_ from **(a)** poor adhesion of bare CF for the untreated sample (0 kV), to **(b)** sporadic PC adhesion with aggregated CF at 150 kV, to **(c)** high consolidation of CFs by the PC matrix with a substantially increased PC/CF adhesion area for 200 kV. In Figure 4a, the untreated sample shows a nearly clean fracture with no PC adhesion to CF, i.e., delamination occurs between the CF1 and PC2 plies. In the 150 kV sample of Figure 4b, some CF bundling is evident with the generation of PC adhesion to CF, along with possible CF breakage, indicating a more jagged delamination between CF1 and PC2. The 200 kV sample in Figure 4c apparently shows complete PC adhesion to CF, with high consolidation of PC within the CF network, and no air spaces between the CFs observed.

EDS mappings in Figure 4a–c (right side) show increased O atoms (green) from the PC bonding with CFs (C in red) from untreated to 150 kV, with the most at 200 kV. A reviewer suggested that elemental composition analysis be carried out, but Figure 4 clearly shows increased O atoms adhering to the HLEBI samples supporting enhanced adhesion. HLEBI activation does not change the overall C/O ratio in the samples and SEM observations already confirm that strong adhesion is achieved by HLEBI by surface and bonding interactions.

## 4. Discussion

### 4.1. Penetration Depth, D_th_, by HLEBI

Strengthening by raising the *V*_c_ of the HLEBI can be explained by the increased *D*_th_ into the interlayered [PC]_10_[CF]_9_ thickness. As mentioned earlier, *D*_th_ into the PC1 outer ply for *V*_c_ of 150 kV was calculated to be 138 μm, greater than the 75 μm ply thickness. Therefore, *D*_th_ into the CF1 ply is estimated by first calculating Δ*V*_PC1_ and *V*_150(CF1)_:

Δ*V*_PC1_ = *T*_PC1_/D_thPC1_ × *V*_N2_ = *T*_PC1_*ρ*_PC1_/[66.7 × (*V*_N2_)^2/3^] =(7.5 × 10^−5^ m) × (1200 kgm^−3^)/[66.7 × (*V*_c_ − Δ*V*_Ti_ − Δ*V*_PC1_)^2/3^] = 59.1 kV (10)

*V*_150(CF1)_ = 150 kV − 24.1 kV − 16.9 kV − 59.1 kV = 49.9 kV (11)

Therefore, by Equation (1), using *V*_150(CF1)_ = 49.9 kV, *D*_th_ into the CF1 ply is 25 μm.

Hence, theoretically, the 150 kV HLEBI does not penetrate through the CF1 ply to the CF1/PC2 interface as shown in Table 5. However, the CF with a 6.5 μm diameter and 10 mm length is reported to have decent electrical conductivity of 6.67 × 10^4^ S/m [95], lower than common metals such as annealed Al or 99.99+% Fe at 10^7^ S/m [96], but much higher than that reported for PC at:10^−14^ S/m [97]. Therefore, charge transfer apparently occurs, reaching the CF1/PC2 interface, to raise the *a*_uc_ over untreated.

Similarly, *D*_th_ values are calculated layer by layer for 175, 200, and 225 kV specimens using Equations (10) and (11) in Table 5 along with *V*_x_ values. Table 5 shows that as *V*_c_ is increased from 150 to 175 to 200 kV, after fully penetrating the outer PC1 ply, *D*_th_ into the next deeper CF1 ply thickness increases from 25 to 65 to 111 μm (bold underlined). The successive increase in *a*_uc_ maxing out at 200 kV can be explained by HLEBI activation throughout increasingly high portions of the 139 μm CF1 ply thickness, culminating at 200 kV with 111 μm/139 μm, or 80% of activation, apparently with enhanced charge transfer to the CF1/PC2 interface.

However, at the higher 225 kV HLEBI setting, although Table 5 shows it activates the PC1 ply throughout its 139 μm thickness with *D*_th_ estimated at 164 μm, and 11 μm into the PC2 ply, the *a*_uc_ is reduced. The excess HLEBI can break newly formed bonds at the interface and weaken PC plies by chain severing [66,80].

**Figure 5:**
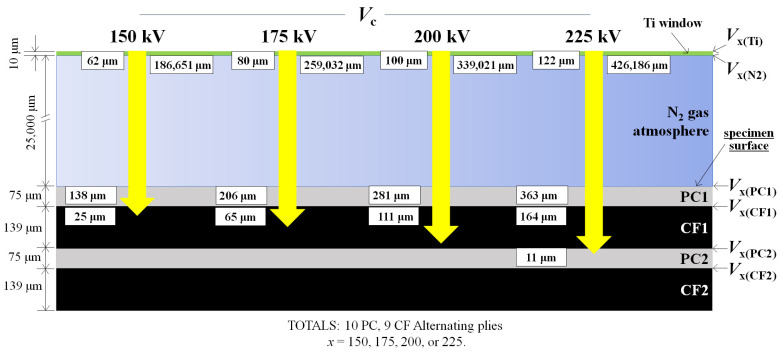
Schematic of HLEBI penetration (yellow arrows) into the surface plies of the interlayered [PC]_10_[CF]_9_ specimen cross-sections for *V*c of 150, 175, 200, and 255 kV data sets, respectively. *D*_th_ and *V*_x_ are shown for each interface. Only the outer 4 plies of one side are shown. Specimens were HLEBI-treated on both sides.

To illustrate the action of HLEBI clearly, Figure 5 is a schematic from Table 5 of successively higher *D*_th_ into the outer plies for the HLEBI data sets. The non-homogeneity of the layered composite structure is depicted, showing PC1, CF1, PC2, and CF2 plies with locations of *V*_x_ and *D*_th_ calculated at each interface, along with those of the Ti window and N_2_. Figure 5 shows the *D*_th_ of 111 μm into the CF1 ply.

**Figure 6:**
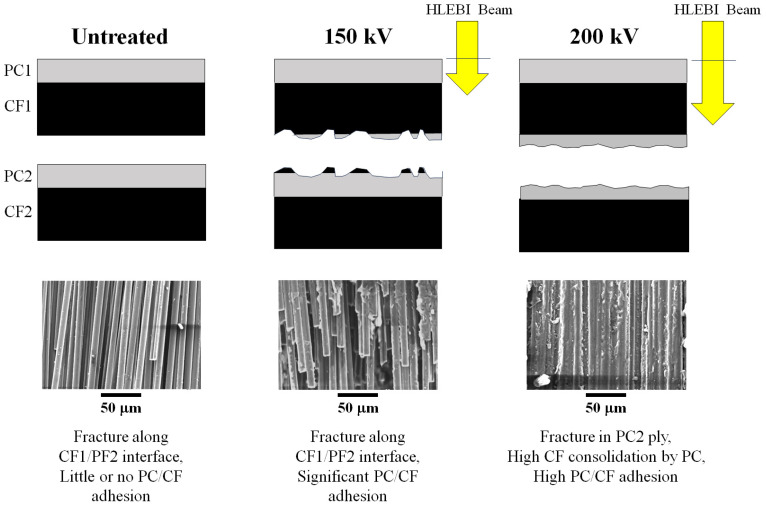
Schematic drawing of fractured CFRPC: untreated, 150 kV, and 200 kV HLEBI samples along with SEM photos.

Figure 6 relates the increasing HLEBI *D*_th_ with the SEM photos from Figure 4 to show the transition in fracture mechanisms to strengthen the [PC]_10_[CF]_9_. First, the untreated sample shows a clean fracture along the CF1/PC2 interface in the form of ply delamination by poor adhesion as evidenced by the bare CF in the SEM photo. However, by applying the 150 kV *V*_c_ setting (*D* = 43.2 kGy), the fracture is transitioned into a more jagged form, partially propagating through the PC matrix and exhibiting significant adhesion along the CF1/PC2 interface to take on the load raising the *a*_uc_. Despite the *D*_th_ of HLEBI (yellow arrows) into the CF1 ply being only about one third (65 μm) of the thickness (139 μm), the CF1/PC2 interface is apparently activated by the charge transfer due to the decent conductivity of CF [96].

On the other hand, when *V*_c_ is increased to 200 kV, *D*_th_ becomes 111 μm, about 80% into the CF1 ply. Here, the increased electron charge will transfer at a stronger level by the conductive CF [95] to the CF1/PC2 ply interface causing increased adhesive bonding sites. The sites act to divert cracks from the PC1/CF2 interface into PC to take on higher load and raise the *a*_uc_. Hence, the 200 kV apparently allows the adhesive force between PC and CF to be greater than the cohesive force in the PC itself. The HLEBI with *V*_c_ of 200 kV thus diverts the fracture from the CF1/PC2 interface to the PC2 ply to increase *a*_uc_ higher than that of the 150 kV or the untreated. The SEM photos support that the 200 kV allows higher generation of PC/CF adhesion surface area than 150 kV. However, 225 kV (not pictured) results in the weakening of the composite due to excess DB generation.

### 4.2. XPS C1s and O1s Results

**Figure 7:**
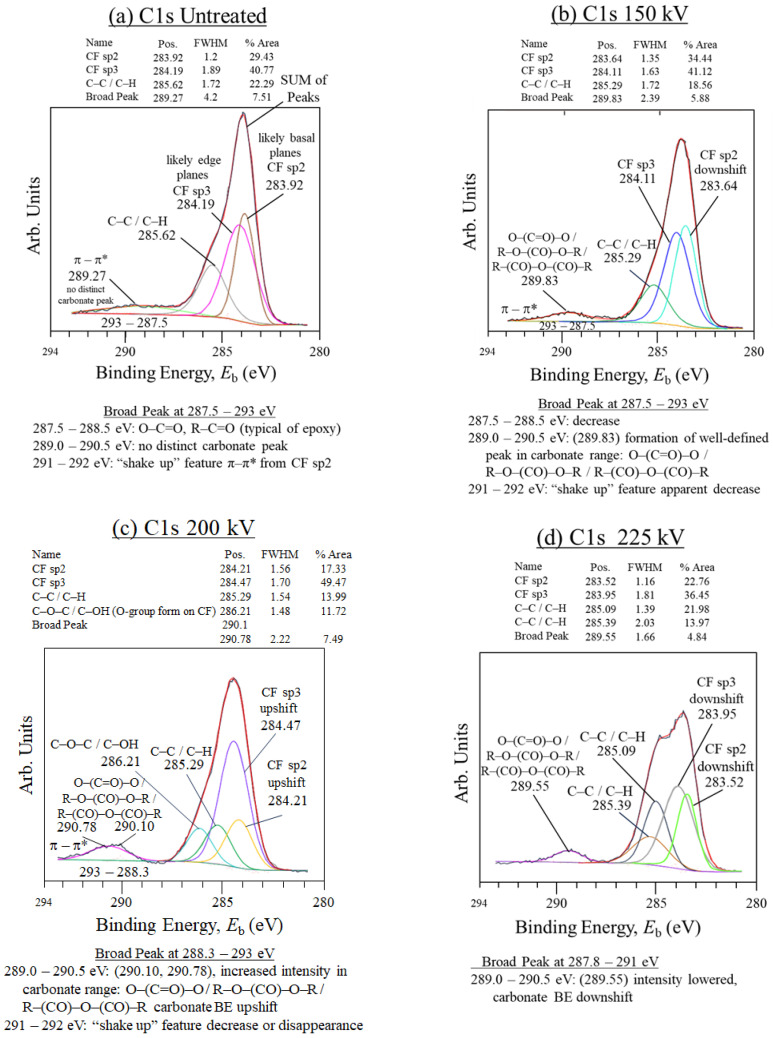
(**a**–**d**) XPS C1s scans of CF in the specimen fracture area. “Downshift” and “upshift” refer to shift in BE of CF sp2 and sp3 as HLEBI *V*_c_ is increased.

**Figure 8:**
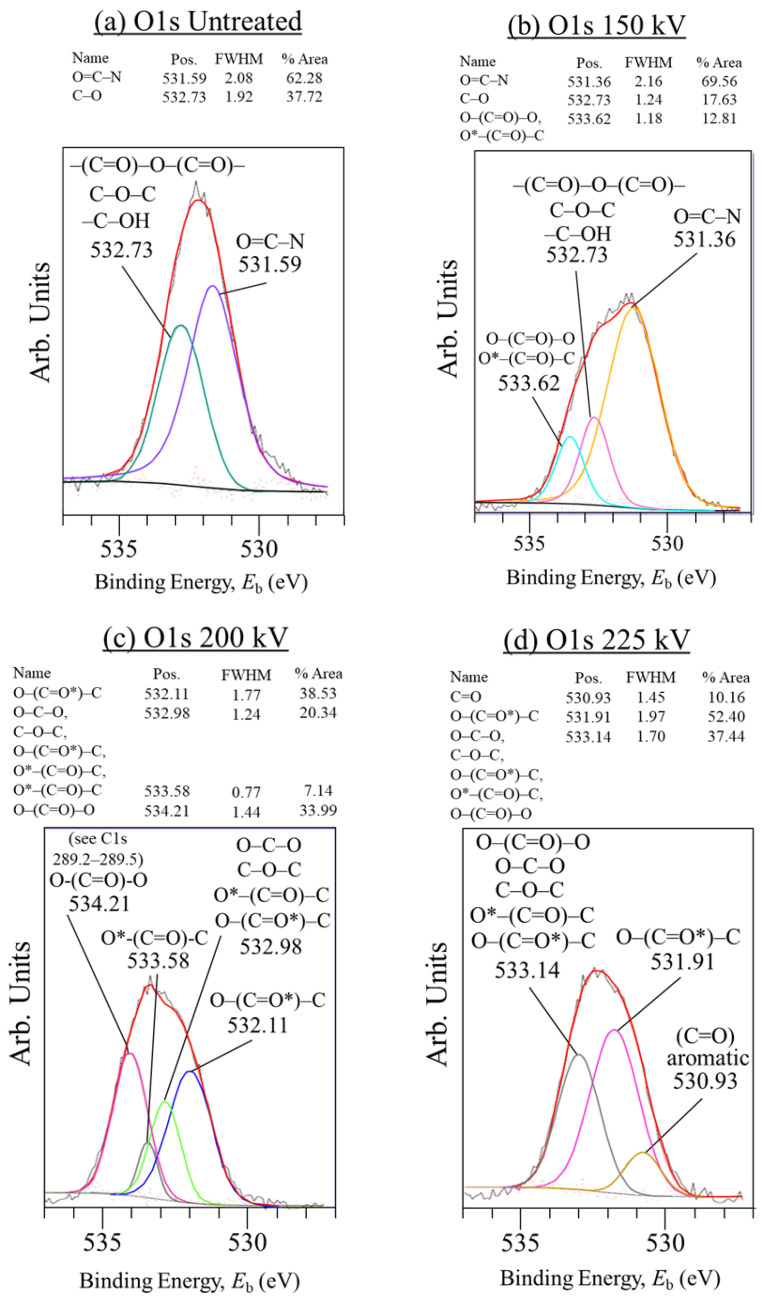
(**a**–**d**) XPS O1s scans of CF in the specimen fracture area. Asterisks (*) designate XPS peak assignments.

The reviewers suggested that in addition to SEM and EDS, XPS, Fourier Transform Infrared (FTIR) spectroscopy, and/or Raman characterizations should be included to explain the interfacial interactions. Therefore, XPS scans were carried out. The results in Figure 7a–d and Figure 8a–d indicate that applying 200 kV HLEBI increases PC adhesion with CF to increase the *a*_uc_ in the [PC]_10_[CF]_9_ samples. Figure 7a–d shows the C1s carbonate O–(C=O)–O and ester O–(C=O)–R binding energy (*E*_b_ or BE) peak generation at 289 to 292 eV to be non-existent in untreated; well-defined at 150 kV; and increased in intensity at 200 kV, after which a reduction was observed at 225 kV. Moreover, the 200 kV yielded the largest area sp^3^ peak at 49.5%, and lowest sp^2^ at 17.3%, signifying an increase in graphitic edge planes in the CF structure apparently as DBs for increased adhesion sites to PC. Downshifts and upshifts in binding energy (*E*_b_) were observed in sp^2^ and sp^3^ peaks and can apparently be attributed to DB generation and dissipation in CF by the HLEBI. Electron spin resonance (ESR) results of 170 kV HLEBI-treated CF showed that naturally occurring DBs existing in untreated CF by peak generation. With an increasing dose, the peak reduces at 43.2 kGy, then increases at 129 kGy and above [81].

C1s scans in Figure 7a,b show that for the untreated sample, a π-π* shake up peak from sp^2^ bonds is evident between about 288 and 293 eV as a flat shape with no distinct carbonate peak. However, at 150 kV there appears the formation of a well-defined carbonate peak at 289.83 eV of O–(C=O)–O and R–O–(C=O)–O–R, along with variation in R–(C–O)–O–(C–O)–R indicating increased PC adhesion to CF. This phenomenon is most prominent in the 200 kV sample with the peaks upshifted to 290.10 and 290.78 eV indicating larger carbonate and related groups for yet higher PC adhesion to CF. Also, for the 200 kV sample, the π-π* peak apparently disappears or is mostly overlapped by the carbonate peaks.

Finally, compared with 200 kV, at 225 kV, the carbonate peak is reduced, apparently from severed PC chains from the excess *V*_c_ setting: full width at half maximum (FWHM) is decreased from 2.22 to 1.66; and % area is decreased from 7.49% to 4.84%. A portion of the emissions at 290.0–291.0 eV can possibly be from trace CF2 oil cleaner. 

Figure 8a–d shows that for the O1s scan, the 200 kV yielded the largest area O–(C=O)–O peak at 34% at the high BE of 534.2 eV, indicating maximum PC adhesion to CF. In contrast, the 150 and 225 eV plots do not appear to have a definite O–(C=O)–O peak, but peaks with mixed groups at lower BE in the 532 to 533 eV range. In addition, the untreated and 150 kV samples exhibited O=C–N peaks at 531.59 and 531.56 eV, respectively. This indicates hardening agent in epoxy sizing remaining on the CFs, in turn confirming the presence of epoxy sizing. Although the 150 kV HLEBI apparently does not remove the sizing, the 200 and 255 kV do, as evidenced by the disappearance of the O=C–N peaks. At the higher 225 kV, the amount of O–(C=O)–O is reduced into ester and other groups, evidence of excess bond severing by the higher *V*_c_ setting, resulting in the lower *a*_uc_ at *P*_f_ of 0.50 of 149 kJm^−2^.

Peaks were designated according to classifications in the literature for C1s [98,99,100], O1s [98,101], sp^2^ and sp^3^ [102], and epoxy [98,100]. Together, the C1s and O1s scan results are consistent with the SEM, that 200 kV HLEBI (at 43.2 kGy dose) is the optimum *V*_c_ out of those tested to increase bonding at the PC/CF interface, raising the impact strength of the [PC]_10_[CF]_9_ samples.

## 5. Conclusions

As far as the authors know, up to now there have been no or few studies on HLEBI to CFRPC found outside of our research group. Experimental results showed that when HLEBI cathode potential is fine tuned to 200 kV at a 43.2 kGy dose to both sides of the finished specimens, the impact strength of an interlayered CFRPC could be increased by 47% over untreated. Increasingly higher settings of 150, 175, and 200 kV successively increased *a*_uc_ at median-*P*_f_ of 0.50 from 118 kJm^−2^ for untreated to 128, 155, and 173 kJm^−2^, respectively. However, the higher 225 kV *V*_c_ setting resulted in a decrease in *a*_uc_ at *P*_f_ of 0.50 to 149 kJm^−2^; therefore, 200 kV appears to be optimum for the cathode potentials tested. SEM showed substantial adhesion of PC to CF by the 200 kV. While XPS C1s and O1s scans of CF in the fracture area detected no carbonate groups for the untreated, carbonate groups were detected for all HLEBI samples, with the highest percentage area at 200 kV.

Specific applications of the CFRPS can include light load-bearing components in airplanes, those of racing car parts, bicycle frames, arrows and crossbow parts in archery, tennis racquets, fishing rods, tent poles, and walking sticks. Other applications can include laptop and cell phone cases, electrical components, due to conductivity and shielding, and musical instruments. In addition, with the recyclability of PC, uses for dwelling on the Moon or Mars are recommended to overcome any lack of resources.

For this research, although detailed potential future research directions are proprietary, they should include tests of tensile and bending, along with temperature, aging studies, microplastic emissions, and life cycle assessment (LCA).

## Figures and Tables

**Table 1:** Ultimate tensile strength (UTS) and its strain of untreated CFRTS (epoxy) [70], CFRPC, along with HLEBI-treated CFRPC [68,69]. Values are calculated according to CF containing portion of specimen cross-section by rule of mixtures [68,69].

Specimen	Epoxy CFRTS	CFRPC	HLEBI-Treated CFRPC
UTS (*σ*_b_: MPa)	413	95.0	290
Strain at *σ*_b_ (*ε*_b_)	0.032	0.015	0.018

**Table 2:** Studies on strengthening CFRPC with HLEBI [66,81,82].

Material	*V*_c_ (kV)	*D* (kGy)	Location	Result
[PC]_4_[CF]_3_	170	216	all CF plies	impact *a*_s_ at *P*_f_ = 0 inc. 6%
[PC]_4_[CF]_3_	170	216	all CF plies	bending *σ*_b_ at *P*_a_ = 0.50 inc. 25%
[PC]_10_[CF]_9_	250	86 to 172	finished surfaces	impact *a*_uc_ at *P*_a_ = 0.50 inc. up to 25 to 30%
[PC]_10_[CF]_9_ (this study)	150, 175, 200, 225	43.2	finished surfaces	impact *a*_uc_ at *P*_a_ = 0.50 inc. up to 47% (200 kV)

**Table 3:** *V*_c_ settings investigated with number of specimens (*D* = 43.2 kGy).

*V*_c_ (kV)	0 (Untreated)	150	175	200	225
Number of Specimens	5	5	5	5	5

**Table 4:** Estimated *V*_x(PC1)_ (kV) at PC1 ply outer surface of sample from *V*_c_ setting and dropped potentials (Δ*V*_Ti_ and Δ*V*_N2_) across Ti window and N_2_ atmosphere of the HLEBI processor.

Cathode Potential (*V*_c_: kV)	Dropped Potential		Surface Electrical Potential at PC1 (*V*_x(PC1)_: kV) (*x =* 150, 175, 200, or 225)	Calculated Penetration Depth into PC1 Ply (*D*_th_: μm)
	In Ti-Window (Δ*V*_Ti_: kV)	In N_2_ Gas Atmosphere (Δ*V*_N2_: kV)		
150	24.1	16.9	109.0	138
175	21.8	14.8	138.5	206
200	19.9	13.3	166.8	281
225	18.4	12.1	194.5	363

**Table 5:** Thicknesses, densities, along with *V*_x_ and *D*_th_ values at each *V*_c_ setting for each successive layer into specimen thickness according to Equations (10) and (11). (HLEBI dose is 43.2 kGy in all cases.)

	Thickness	Density	Voltage at Top Surface of Layer (*V*_X_: kV)/ Penetration Depth from Top Surfaces (*D*_th_: μm)							
Material	*T* (μm)	*ρ* (kg/m^3^)	*V*_c_ = 150 kV		175 kV		200 kV		225 kV	
(layer)			*V*_150_	*D*_th_	*V*_175_	*D*_th_	*V*_200_	*D*_th_	*V*_225_	*D*_th_
Ti window	10	4540	150	62	175	80	200	100	225	122
N_2_	25,000	1.13	125	186,651	153	259,032	180	339,021	207	426,186
PC1	75	1200	109	138	138	206	167	281	194	363
CF1	139	1800	50	**25**	88	**65**	122	**111**	154	**164**
PC2	75	1200	0	0	0	0	0	0	24	**11**
CF2	139	1800	0	0	0-	0	0	0	0	0

## Data Availability

The original contributions presented in this study are included in the article. Further inquiries can be directed to the corresponding authors.
